# Age-related neuroinflammation and changes in AKT-GSK-3β and WNT/ β-CATENIN signaling in rat hippocampus

**DOI:** 10.18632/aging.100853

**Published:** 2015-12-06

**Authors:** Ana Maria Marques Orellana, Andrea Rodrigues Vasconcelos, Jacqueline Alves Leite, Larissa de Sá Lima, Diana Zukas Andreotti, Carolina Demarchi Munhoz, Elisa Mitiko Kawamoto, Cristoforo Scavone

**Affiliations:** ^1^ Department of Pharmacology, Institute of Biomedical Sciences, University of São Paulo, 05508-900 São Paulo, Brazil

**Keywords:** NF-κB, AKT, β-CATENIN, GCs, GSK-3β

## Abstract

Aging is a multifactorial process associated with an increased susceptibility to neurodegenerative disorders which can be related to chronic inflammation. Chronic inflammation, however, can be characterized by the persistent elevated glucocorticoid (GCs) levels, activation of the proinflammatory transcription factor NF-кB, as well as an increase in cytokines. Interestingly, both NF-кB and cytokines can be even modulated by Glycogen Synthase Kinase 3 beta (GSK-3β) activity, which is a key protein that can intermediate inflammation and metabolism, once it has a critical role in AKT signaling pathway, and can also intermediate WNT/β-CATENIN signaling pathway. The aim of this study was to verify age-related changes in inflammatory status, as well as in the AKT and WNT signaling pathways. Results showed an age-related increase in neuroinflammation as indicated by NF-кB activation, TNF-α and GCs increased levels, a decrease in AKT activation and an increase in GSK-3β activity in both 12- and 24- month old animals. Aging also seems to induce a progressive decrease in canonical WNT/β-CATENIN signaling pathway once there is a decrease in DVL-2 levels and in the transcription of *Axin2* gene. Little is known about the DVL-2 regulation as well as its roles in WNT signaling pathway, but for the first time it was suggested that DVL-2 expression can be changed along aging.

## INTRODUCTION

A very recent study highlight the fact that neuro-degenerative disorders are responsible for disability and death of more than 30 million people worldwide [1]. Thus, some theories have been proposed to explain the aging process, as the theory of random accumulation of molecular damage in which cancer and aging should have similar origins although they seem opposite processes [2] and the hyperfunction theory that was first characterized by cellular hypertrophy followed by atrophy that try to explain the outgrowth of diabetes, cardiovascular disorders and chronic inflammation along the process [3, 4].

Aging can be considered as a condition in which the organism is not able to balance the effects of oxidative stress and inflammation, leading to an increased susceptibility of neurons to death, coupled with a progressive decline in signaling molecules which can result in a decrease in motor and cognitive performances, especially memory [5]. Changes in learning and memory process reflect compromised hippocampal function and can really affect a personal quality of life and the ability to maintain an independent lifestyle [6, 7].

However, brain aging has been considered a multifactorial process where a significant neuronal loss does not contribute by itself to an age-related cognitive decline [8, 9]. Nearly 40 years ago, neuroscientists began to correlate metabolic deregulation with the pathophysiology of neurological and psychiatric disorders due to Professor John P. Blass studies [10]. Since then, many proteins have been identified as crucial to intermediate the crosstalk between different signaling pathways involved either in metabolism and neuro-degenerative process, such as GSK-3β, which has been considered a pro-inflammatory protein in addition to its fundamental role in insulin signaling pathway [10, 11].

In agreement with the hyperfunction theory, many hallmarks of aging have emerged. It is well known that human aging is accompanied by increased serum levels of pro-inflammatory cytokines, such as IL-1, IL-6 and TNF-α, what seems to be related to the immuno-senescence process [4–7, 12], as well as NF-кB signaling pathway [12–14], that can be considered as a key molecular system for brain inflammation leading to metabolic syndrome [15].

NF-кB is constitutively expressed in the cytoplasm where it is retained by a family of inhibitory molecules termed inhibitor κB (IκB) [16]. NF-κB activity is attributed to the Rel/NF-κB protein family forming homo- and heterodimers through the combination of the subunits p65 (or RelA), p50, p52, c-Rel and RelB. This transcription factor can be activated by lipopolysaccharide (LPS), cytokines such as tumor necrosis factor (TNF-α) and interleukin 1β (IL-1β), and reactive oxygen species [17, 18]. LPS, IL-1β, TNF-α, and reactive oxygen species all induce NF-κB by activating IκB kinases which phosphorylate IκBα, leading to its polyubiquitination and degradation at the proteasome 26S [19], allowing NF-κB to migrate to the nucleus and to modulate the transcription of its target genes.

However, in the presence of glucocorticoids (GCs), the nuclear translocation of NF-κB can be prevented because GCs induce IκBα expression [20, 21], and can direct interact with the NF-κB p65 subunit, thereby blocking NF-κB-DNA-binding activity [22–24]. In the presence of a chronic inflammatory process, glucocorticoids (GCs), that are well-known to be anti-inflammatory and immunosuppressive, can have pro-inflammatory effects in the brain [25, 26].

Another very important hallmark of aging is the mTOR signaling pathway that has PI3K/AKT upstream [2, 12]. AKT (also known as protein kinase B) is a serine/threonine kinase that is believed to promote cell viability in many different cell types [27, 28], including neurons, by phosphorylating and thus inactivating downstream targets such as the Ser/Thr kinase GSK-3β, forkhead transcription factor, pro-apoptotic Bcl-2 family members, and caspase-9 [29, 30]. AKT is also known to regulate non-canonical NF-кB p52 processing by increasing the activity of IKKα [31].

Glycogen synthase kinase-3 (GSK-3) was first identified by its glycogen synthase phosphorylating activity [32] and some years later was described in the Central Nervous System (CNS) as a kinase of Tau protein [33, 34]. The regulation of GSK-3 activity can be summarized in four key mechanisms which consist in the regulation of its phosphorylation state, its subcellular localization, the formation of protein complexes containing GSK-3, and the phosphorylation state of its substrates [10]. Regarding phosphorylation, *GSK-3 activity* is *inhibited through phosphorylation* of *serine 21* in α isoform and of *serine 9* in *β-isoform* [35]. When phosphorylated in the Ser9 residue, GSK-3β is inactivated, increasing the stabilized unphosphorylated state of β-CATENIN [36]. Thus, AKT/GSK-3β/β-CATENIN signaling plays a critical role in neuroinflammation and apoptosis of neurons in several models of neurodegeneration [37, 38].

Furthermore, β-CATENIN is a key component of the canonical WNT signaling pathway, acting as a transcriptional coactivator [37]. WNT proteins are secreted glycoproteins that bind to the cell surface Frizzled (Fz) receptor family and play an important role during development and in adult tissue homeostasis, but little is known about changes in β-CATENIN in senescence [39]. WNT signaling pathway can be classified as canonical, when β-CATENIN is an important effector of the pathway, and non-canonical, when signaling happens independent of β-CATENIN activation [40].

In the absence of a WNT stimulus, cytoplasmic β-CATENIN is phosphorylated and degraded by proteasome [41, 42], due to the action of two kinases, GSK-3 and Casein kinase 1 alpha (CK1α), localized in two multidomain scaffolding proteins called AXIN and Adenomatous polyposis coli (APC), also known as destruction complex [42]. When WNT protein binds to the Fz receptor, it recruits the co-receptor LRP5/6 [43], and both Dishevelled (DVL) and AXIN proteins, which results in inhibition of β-CATENIN phosphorylation and thus ensuing its stabilization [42]. The stabilized β-CATENIN can also translocate to the nucleus where, together with Tcf/Lef proteins, it can control the transcription of specific genes like *Axin 2*, which acts as a negative-feedback regulator of WNT signaling [44].

Recent studies also suggest an important role for DVL shaping neuronal projections [45] and in neural connectivity establishment [46], in a model of embryonic development of *Caenorhabditis elegans* and in *Schmidtea mediterranea, respectively* [47, 48]*.* These actions of DVL can only be possible because DVL is a common intracellular mediator of canonical and non-canonical WNT signaling pathway, interacting with different proteins due to three conserved regions that are known as the DIX, PDZ and DEP domains [49]. Through these interactions, DVL can be found in various cellular compartments, as in complex with microtubules and actin cytoskeleton [50, 51] and in the nucleus, where interaction with c-Jun and β-CATENIN followed by formation of the stable β-CATENIN/TCF complex was also demonstrated [52–54].

Therefore, the present manuscript shows an age-related decrease of AKT signaling leading to an increase in the activation of the proinflammatory protein GSK-3β as well as an increase in neuroinflammation signaling associated to NF-κB, TNF-α and serum GCs. Finally, our results suggest that aging process is associated to a decrease in WNT/β-CATENIN signaling pathway that can be related to a decrease in DVL-2 levels.

## RESULTS

### Age-Induced changes in NF-κB activity, TNF-α and IL-10 levels in hippocampus and serum GCs in rats

**Figure 1:**
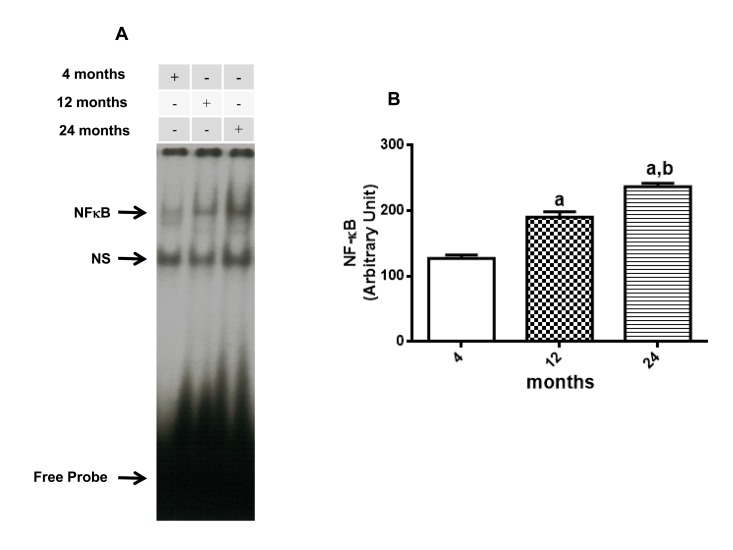

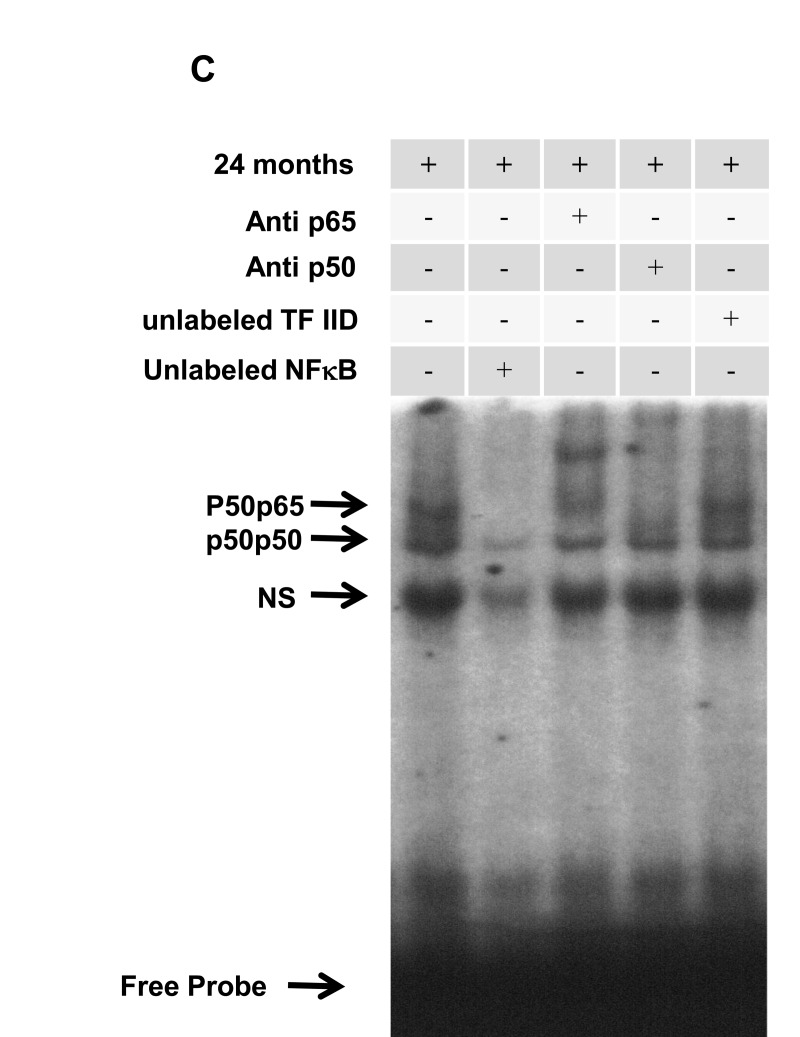
Age-related NF-κB activation in rat hippocampus (**A**) NF-κB activation in hippocampus of 4-, 12- and 24-month-old rats. The positions of both protein/DNA complexes observed are indicated as NF-κB (p50/RelA and p50/p50). NS represents nonspecific binding. The localization of free probe is also indicated. Results are representative of three experiments. (**B**) Densitometric analysis (arbitrary units) of the bands p50/RelA and p50/p50 presented in panel A. Results are expressed as mean + S.E.M. from three individual experiments. ANOVA followed by Newman-Keuls. (a) p<0.05 vs. 4-month-old basal sample, (b) p<0.05 vs. 12-month-old basal sample. (**C**) Competition studies were performed using nuclear extract (15 μg) from hippocampus of 24-month-old rats in the absence or presence of unlabeled specific (NF-κB consensus sequence, 20-fold molar excess) or non-specific oligonucleotide (TFIID consensus sequence, 20-fold molar excess), as indicated. Supershift assays were performed with the same nuclear extract (15 μg) incubated in the absence or presence of antibodies against subunits p50 (1:20 dilution) and p65 (1:20 dilution) as indicated. Antibodies were added 20 min prior to addition of the radiolabeled NF-κB consensus oligonucleotide. The positions of specific NF-κB/DNA binding complexes p50/p50 and p50/p65 are indicated. NS represents nonspecific binding. The localization of the free probe is also indicated.

To evaluate age-related changes in NF-κB activity in rat hippocampus, Electrophoretic Mobility Shift Assay (EMSA) were performed. Results suggest that age induced a progressive activation of NF-κB (Fig. 1A). Nuclear extracts from the tissues of 4-, 12- and 24-month-old animals presented a similar pattern of three DNA/protein complexes (Fig. 1C). Respective densitometric analyses are shown (Fig. 1B).

The complexes p50/RelA and p50/p50 (represented as NF-κB band in the figure) were displaced by an excess of unlabeled NF-κB, but not by TFIID double stranded oligonucleotide consensus sequence, demonstrating the specificity of NF-κB/DNA binding interaction (Fig. 1C). The complex non-specific (NS) was less efficiently displaced by unlabeled P^32^-NF-κB probe (Fig. 1C). Super-shift analysis indicated that the antibody against the RelA subunit was able to shift the DNA/protein interaction observed in p50/RelA. The antibody against the p50 subunit shifted complex p50/RelA and induced a partial decrease in complex p50/p50. The presence of antibodies against the p52 and c-Rel subunits did not affect the DNA-protein complexes (data not shown). Taken together, these results indicated that p50/RelA heterodimers and p50/p50 homodimers were present in NF-κB/DNA complex. Complex non-specific (NS) was not displaced by the antibodies and so it was not con-sidered to be a member of the NF-κB family (Fig. 1C).

**Figure 2:**
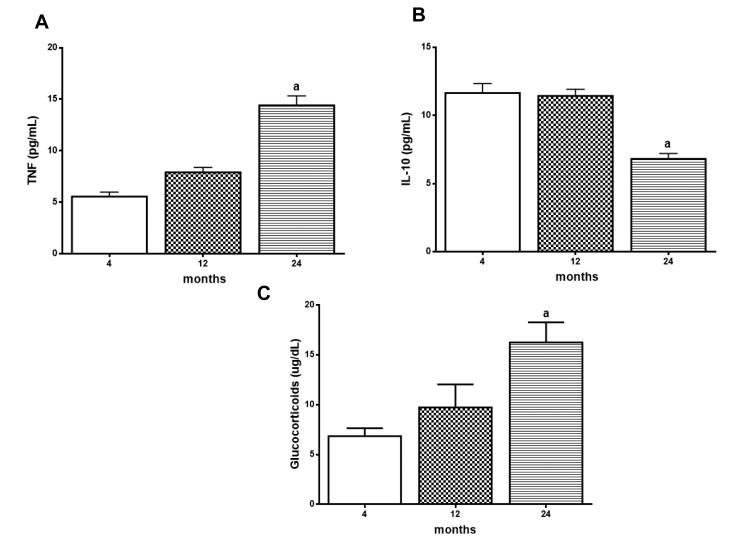
Age related changes in TNF‐α, IL‐10 and Glucocorticoid (GC) levels Levels of hippocampal TNF‐α (**A**) and IL‐10 (**B**) and serum glucocorticoids (**C**) were measured by ELISA kits. Results are expressed as mean ± S.E.M. from three individual experiments (n= 5‐10 for each experimental group). ANOVA followed by Newman‐Keuls. (a) p<0.05 vs. 4‐month‐old basal sample.

To evaluate the age effect in the NF-κB target genes expression, TNF-α and IL-10 levels were measured in rat hippocampus. Although there is an age-related progressive tendency of increase in the levels of TNF-α, the significant increase was only detected in 24-month-old animals (Fig. 2A). The results also showed a decrease in IL-10 levels at 24 months when compared to the groups of 4- and 12-month-old animals (Fig. 2B).The same results were observed in serum GC levels as measure of corticosterone which showed a significant increase in 24 month-old animals (Figure 2C).

### Age-related changes in AKT-GSK-3β signaling pathway

**Figure 3:**
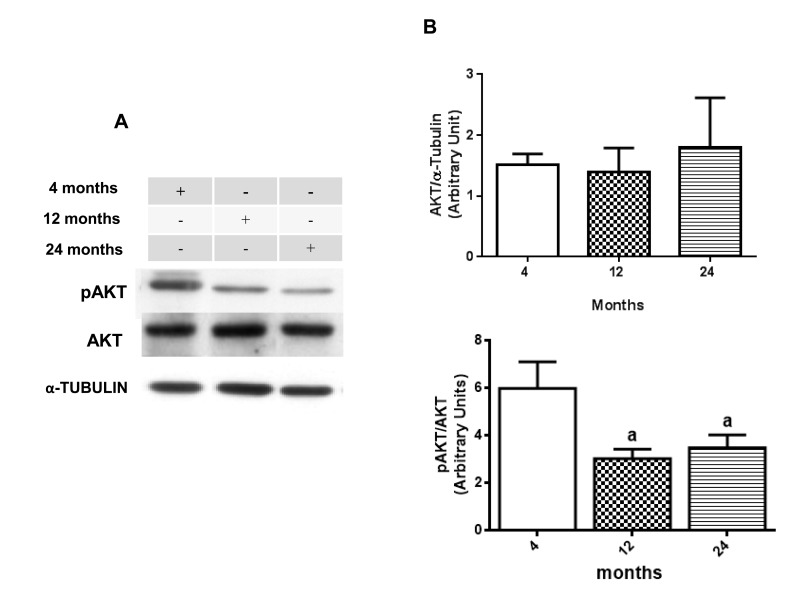
Changes in the pAKT/AKT ratio in hippocampus of 4-, 12- and 24-month-old rats (**A**) Representative Western blotting autoradiographs of cytosolic AKT/α-TUBULIN and pAKT/AKT. (**B**) Densitometric analysis (arbitrary units) of the ratio of data presented in A panel are shown. No significant difference in densitometric analysis of AKT/α-TUBULIN values was found between all groups. Results are expressed as mean ± S.E.M. from three individual experiments. ANOVA followed by Newman-Keuls. (a) p<0.05 vs. 4-month-old basal sample.

**Figure 4:**
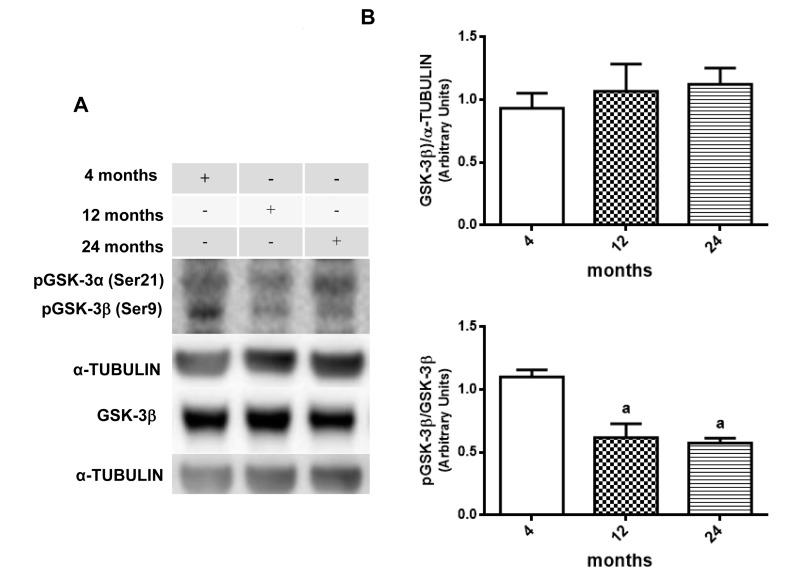
Changes in the pGSK-3β/GSK-3β ratio in hippocampus of 4-, 12- and 24-month-old rats (**A**) Representative Western blotting autoradiographs of cytosolic GSK-3β/α-TUBULIN and pGSK-3β/GSK-3β. (**B**) Densitometric analysis (arbitrary units) of the ratio of data presented in panel A is shown. No significant difference in densitometric analysis of GSK-3β/α-TUBULIN values was found between all groups. Results are expressed as mean ± S.E.M. from three individual experiments. ANOVA followed by Newman-Keuls. (a) p<0.05 vs. 4-month-old basal sample.

Once GSK-3β could be considered as a potential therapeutic target for diabetes and neurological disorders due to its role in insulin pathway substrates and inflammation process [55, 56], western blot assays were performed to verify the presence of some age related changes in AKT and GSK-3β activity. Results suggest an age-related decrease in phosphorylation ratio for both AKT and GSK-3β. When compared, phosphorylated AKT at Ser 473 versus un-phosphorylated AKT, it is possible to observe a significate decrease in 12- and 24-month-old rats compared to 4 month-old animals, although there is no difference in total AKT expression among the groups (Fig. 3A and 3B). The same was observed in the rate of phosphorylated GSK-3β on Ser9 versus total GSK-3β, which showed a similar decrease at 12- and 24-month-old animals when compared to 4-month-old animals in hippocampus (Fig. 4A and 4B).

### Age-related decrease in DVL-2 and WNT/β-CATENIN signaling pathway

It is well known that GSK-3β can also participate in WNT signaling pathway phosphorylating the β-CATENIN, and addressing this protein to degradation via proteasome in the absence of WNT activation. Although WNT activation does not lead to Ser9 phosphorylation, there is a pool of GSK-3β that is present in a large protein complex, called destruction complex, which includes, in addition to β-CATENIN and GSK-3β, the proteins DVL, AXIN and CK1 [42]. Furthermore, WNT/β-CATENIN signaling can induce the transcription of *Axin2* gene, which completes a negative feedback acting as a negative regulator of the signaling pathway [57].

**Figure 5:**
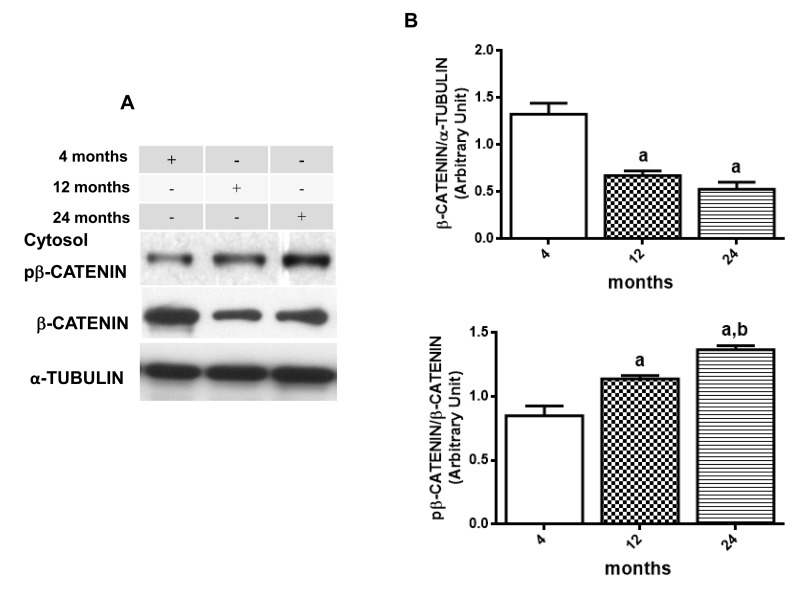

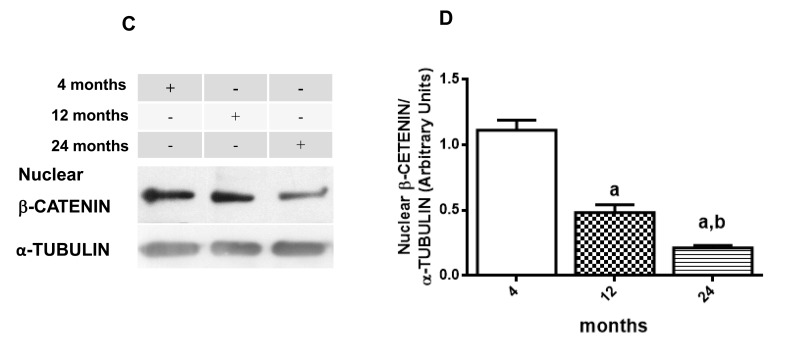
Changes in β-CATENIN signaling in hippocampus of 4-, 12- and 24-month-old rats (**A**) Representative Western blotting autoradiographs of cytosolic βCATENIN/αTUBULIN and pβCATENIN/βCATENIN. (**B**) Densitometric analysis (arbitrary units) of βCATENIN/αTUBULIN and pβCATENIN/βCATENIN ratios of data presented in A panel are shown. Results are expressed as mean ± S.E.M. from three individual experiments. ANOVA followed by Newman-Keuls. (a) p<0.05 vs. 4-month-old basal sample, (b) p<0.05 vs. 12-month-old basal sample. (**C**) Representative Western blotting autoradiographs of nuclear β-CATENIN and α-TUBULIN. (**D**) Densitometric analysis (arbitrary units) of β-CATENIN/α-TUBULIN ratio of data presented in C panel is shown. Results are expressed as mean ± S.E.M. from three individual experiments. ANOVA followed by Newman-Keuls. (a) p<0.05 vs. 4-month-old basal sample, (b) p<0.05 vs. 12-month-old basal sample.

**Figure 6:**
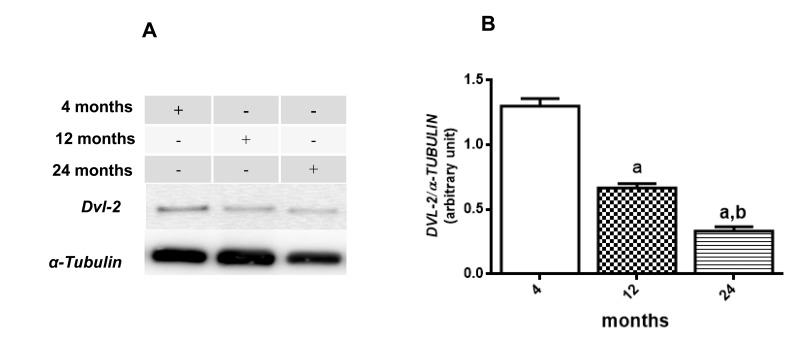
Changes in the DVL-2 levels in hippocampus of 4-, 12- and 24-month-old rats (**A**) Representative Western blotting autoradiographs of cytosolic DVL-2 and α-TUBULIN. (**B**) Densitometric analysis (arbitrary units) of DVL-2/α-TUBULIN ratio of data presented in A panel are shown. Results are expressed as mean ± S.E.M. from three individual experiments. ANOVA followed by Newman-Keuls. (a) p<0.05 vs. 4-month-old basal sample, (b) p<0.05 vs. 12-month-old basal sample.

**Figure 7:**
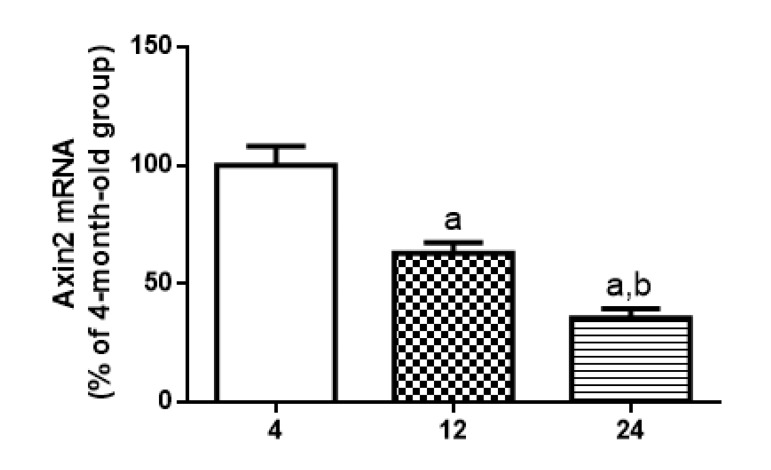
Age-related changes in *Axin2* mRNA levels in hippocampus of 4-, 12- and 24-month-old rats Levels of mRNA encoding *Axin2*, measured by quantitative RT-PCR in samples from hippocampus. Results are expressed as percentage of 4-month-old group ± S.E.M., n = 5 for each experimental group. ANOVA followed by Newman-Keuls. (a) p<0.05 vs. 4-month-old basal sample, (b) p<0.05 vs. 12-month-old basal sample.

Following the data above, immunoblotting was done to verify β-CATENIN phosphorylation status and DVL2 cytosolic expression. Results suggest a decrease in WNT/β-CATENIN activity since the basal rate of cytosolic phosphorylation of β-CATENIN in relation to total β-CATENIN is increased along the aging process (12 month-old increases 133.8 % and 24 month-old increases 160.8 % compared to the values detected at 4 months) whereas levels of nuclear β-CATENIN seems to decrease in 12 and 24 months old compared to young rats (53,3% and 26% of 4 month old ratio, respectively) (Fig. 5A 5C and 5D). In addition, total DVL-2 levels were decreased in cytosol to 49% at 12 months and 17% at 24 months, when compared to the values obtained in 4-month-old animals (Fig. 6A and 6B). Finally, a quantitative RT-PCR assay was performed to detect levels of *Axin2* transcription. Results suggest a decrease in transcription of *Axin2* gene along the aging process (Fig. 7).

**Figure 8:**
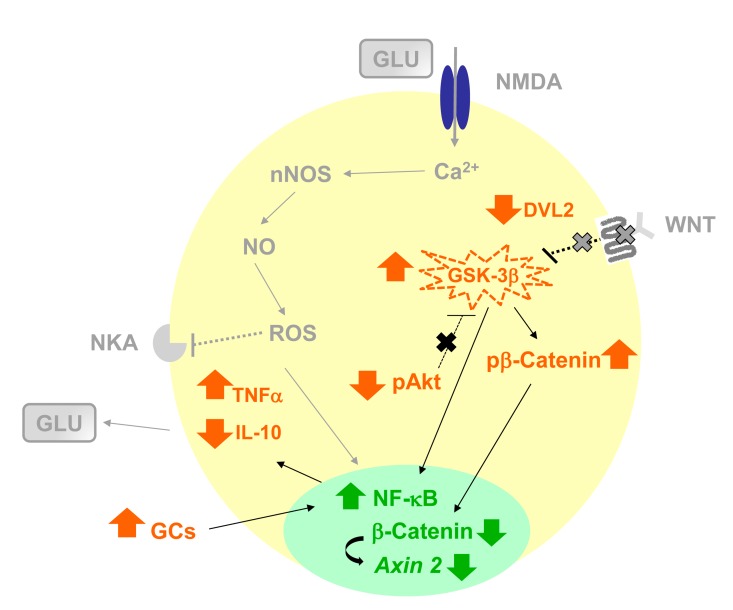
Schematic representation of the hypothetical influence of age-related neuroinflammation and changes in AKT-GSK-3β and DVL-2/ β-CATENIN signaling in rat hippocampus, increasing susceptibility to neurodegenerative process All data showed in present manuscript are in color pattern: orange for protein expression in cytosol, except for GSK-3β that has activity level represented, and green for nuclear expression of transcription factors and mRNA. All hypothetical explanations are in grey and were not measured in this paper. The present work showed that age-related increase in neuroinflammation as indicated by NF-кB activation, increase in TNF-α and decrease of IL-10 levels, combined with a basal reduction in the pAKT increasing GSK-3β activity, what can be related to a decrease in DVL-2/β-CATENIN pathway in hippocampus in the presence of high levels of serum GCs, may predispose an effect of specific factor in the manifestation of age-related degenerative disorders. In fact, in response to the neuroinflammatory status and decrease of DVL-2 linked to β-CATENIN pathway in hippocampus, microglia should become activated and high levels of glutamate (GLU) is frequently associated with impaired handling of extracellular GLU by gliotic astrocyte [100–103].

## DISCUSSION

Our results showed an age-related progressive increase in NF-кB activity in rat hippocampus, mediated by p50/RelA heterodimers and p50/p50 homodimers (Fig.1). The first combination of heterodimers can act as a potent transcriptional activator of genes usually activated by synaptic activity and thereby provides a mechanism of signaling that likely plays an important role in neuroinflammatory response; while the homodimer p50/p50 is well known as a stimulus-specific repressor of gene activation, relevant to *modulate* the activation of *inflammatory response genes* [58].

The result presented here is in agreement with previous data which showed an increase of NF-кB activity in a variety of tissues along aging, such as hypothalamus [59], frontal cortex, cerebellum [60], as well as in liver of 30-month-old rats [61]. In addition, inhibition of NF-кB activity seems to lead to a delayed onset of age-related symptoms and pathologies in animal models, and it has been linked with known lifespan regulators [14, 62]. Therefore, the importance of NF-кB signaling has been very well described in inflammaging and its activators could be potential aging biomarkers [63, 64].

Moreover, we found an age-related increase of TNF-α levels, a cytokine that plays a central role in the onset and maintenance of inflammation [65], and a decrease of IL-10 levels, a prototypical anti-inflammatory cytokine [66, 67], in rat hippocampus (Fig. 2). The increase in the ratio TNF-α/IL-10 might be related with the age-related maintenance of NF-кB activation, although the participation of other pathways cannot be ruled out. In addition, it was demonstrated that aging induced an increase in serum GCs levels (Fig. 2). It is well established the association of GCs with anti-inflammatory effects. However, in some circumstances as chronic inflammatory process observed in aging, GCs can enhance instead of reducing inflammation [68], which could justify the increased levels of TNF-α observed and the reduction of IL-10. Short-term GCs, as with immune activation, can be beneficial, whereas long-term GCs and prolonged or exaggerated inflammation may result in adverse consequences [69].

In fact, recent data from our laboratory have shown that nephrectomy-induced cognitive impairment in rats seems to be related to an *increase in GCs levels in* cerebrospinal fluid (CSF) and *TNF-α/IL-10 ratio compared to the Sham group* [70]*. Furthermore,* chronic unpredictable stress (CUS) potentiated lipo-polysaccharide (LPS)-induced NF-кB activation and pro-inflammatory cytokine expression in the frontal cortex and hippocampus, but not in hypothalamus of rats. The pro-inflammatory effects of stress were mediated by GC receptors since pretreatment with the GR antagonist RU-486 reverted the stress effects on NF-кB potentiation [25].

Therefore, GCs may have variable effects on age-related inflammatory processes, dampening inflammation in some circumstances where inflammatory stimulus is weak, whilst enhancing inflammation in other circumstances, at least in part via the sensitization of microglia reactivity, increasing glutamate (GLU) release [71] which in turn can induce NF-кB activation [72].

Another hallmark of aging that seems to be one of the most promising therapeutic strategy for neurodegenerative disorders until this moment is the mechanistic target of rapamycin (mTOR) [1, 73]. Moreover, AKT is a well-known component of the mTOR signaling pathway [74], that together with WNT signaling can prevent caspase activation and apoptosis [75]. However during the aging process mTOR signaling is reduced, as well as AKT [45], and WNT/β-CATENIN in hippocampus, as suggested in this manuscript and by other groups, in hippocampal neurons [76] and in human fibroblast cell lineage [77].

Studies from literature have suggested that mis-regulation of canonical WNT signaling can be involved in Alzheimer’s disease (AD) [78, 79] due to the fact that AD patients that carries a mutation in presenilin-1 have very low levels of β-Catenin [80]. An interesting information is that Insulin like growth factor-1 (IGF1) signaling that is mediated via the activation of PI3K and AKT signaling seems to be very important for dendritic growth [76] and to be regulated by WNT/ β-CATENIN signaling [81]. In an elegant approach, Kim et al., have demonstrated that hippocampal neurons treated with 1μM ICG-001, a small molecule that selectively inhibits TCF/ β-CATENIN transcription [82], for 24 hours, has a decrease in AKT phosphorylation and an increase in neuronal degeneration [76]. Similar results were obtained when transgenic mice overexpressing AXIN 2 in hippocampus were analyzed [76].

Considering the results already presented and taking into account the contribution of GSK-3β to the development and progression of many neurological diseases due to its regulation in neuroinflammatory processes [83], it was possible to observe an age-related decrease in the pGSK-3β/GSK-3β ratio in 12- and 24-month-old rats (Fig. 4) accompanied by the decrease in the phosphorylation ratio of the upstream protein AKT (Ser 473) at 24-month-old rat in comparison to the 4-month-old group (Fig. 3). Thus, an increase in GSK-3β activity could be related to the presence of neuroinflammation and mTOR/AKT. In fact, GSK-3β has been linked to the modulation of an assembly of transcription factors, including the activation of NF-κB and inhibition of β-CATENIN [84].

In addition, some studies indicate a possible crosstalk between NF-кB signaling pathway and WNT/β-CATENIN pathway [85]. In the present manuscript, we demonstrated that aging induced a progressive decrease in basal nuclear β-CATENIN expression and an increase in the rate of cytosolic pβ-CATENIN/β-CATENIN (Fig. 5). We also reported for the first time an age-related decrease in total DVL-2 expression in rat hippocampus (Fig. 6), and a decrease in the gene transcription of *Axin2* (Fig. 7), which is a product of the β-CATENIN signaling pathway and a negative feedback regulator of the pathway [44, 57]. Although there is some uncertainty about a functional cross-regulation between these two pathways (NF-кB and β-CATENIN) in hippocampus during aging process, it is well known that both pathways have shown complex roles for pathogenesis of certain age-related diseases [85].

The Dishevelled family proteins (Dvls) are multifunctional intracellular proteins essential for the transduction of WNT signaling [86, 87]. DVL proteins are among the few signaling intermediaries common to both canonical and non-canonical pathways; however, it is only in the canonical one that DVL, in the presence of a WNT stimulus, is recruited to Fz receptor to form a signalosome with AXIN and other proteins to inactivate β-CATENIN destruction complex [88, 89]. Otherwise, some studies have suggested that phosphorylation of DVL can occur in the absence of β-CATENIN stabilization [90] which would not be an appropriate measurement for our question. In summary, aging seems to negatively modulate the DVL-2-activation of β-CATENIN signaling pathway, which is in agreement with a very recent study that suggests physical exercise and environmental enrichment as factors that can attenuate the decrease in WNT signaling pathway along aging process [91].

It is important to emphasize that in recent years WNT signaling has been emerging as an important factor in normal functioning of the hippocampus [92] with growing evidence that its downregulation could be involved not only in cognitive decline associated with normal aging, but even with the physiology of AD [93]. Understanding the molecular mechanisms underlying this downregulation as well as strategies to reverse this decrease would be of fundamental importance.

Previous studies have already suggested that strategies that induce neurogenesis and neuroprotection, such as exercise and reduced caloric intake, could be a potential mechanism to preserve and/or restore brain function during aging and injury [94], especially with respect to inflammatory process [95, 96].

In summary, our results suggest an age-related increase in inflammatory process in rat hippocampus due to the increase in NF-кB activation and TNF-α/ IL-10 ratio. In addition, there is an age-related decrease in DVL-2 levels that is involved in the WNT/β-CATENIN signaling downregulation. Although further studies are necessary to elucidate the mechanisms involved, as well as to understand the crosstalk between these pathways, data suggest that DVL-2 might be a good target to protect the CNS against the deleterious effects of aging.

## MATERIALS AND METHODS

### Animals and tissue preparation

Four-, 12- and 24-month-old male Wistar rats (Biomedical Sciences Institute, University of São Paulo) were kept under 12 h light/dark cycle (lights on at 7:00 A.M.) and allowed free access to food and water. All animals were sacrificed by decapitation (between 9:00 and 11:00 AM) following procedure approved by the Biomedical College of Animal Experimentation (COBEA). All procedures were also approved by the Ethical Committee for Animal Research (CEEA) of the Biomedical Sciences Institute of the University of São Paulo. The brain was immediately removed and immersed in cold PBS. The hippocampus was rapidly dissected, quickly immersed in liquid nitrogen, and stored at −80°C for later use.

### Measurement of TNF-α and IL-10 in hippocampus and serum GCs levels by ELISA kits

Concentrations of TNF-α and IL-10 were measured in 25 μL of extracts from homogenized hippocampus obtained from 4-, 12- and 24-month-old rats by using a conventional enzyme-linked immunosorbent assay (ELISA) kits (eBioscience, San Diego, CA). Briefly, hippocampal tissue was homogenized in a buffer containing 150 mM NaCl, 0.05% Tween-20, 1 mM PMSF, 2.5 ug/mL Antipain, 2.5 ug/mL Leupeptin and 20 mM Tris-HCl (pH 7.5), and protein concentration was measured in each sample. The TNF-α and IL-10 concentrations are expressed as pmol/mL.

Concentrations of GCs were measured in 25 μL of serum from 4-, 12- and 24-month-old rats were quantified by using an enzyme-linked immunoassay (Assay Designs, Ann Arbor, MI). Trunk blood was collected in 15 ml conical tubes and centrifuged at 3,000 rpm for 10 min to obtain serum. GCs titers were assessed by using a competitive enzyme immunoassay kit, following the manufacturer’s instructions.

### Immunoblotting

Electrophoresis was performed using 10% polyacrylamide gel and the Bio-Rad mini-Protean III apparatus. In brief, the proteins present in the hippocampus cytosolic fraction (15 μg) were size-separated in 10% SDS-PAGE (90 V). The proteins were blotted onto a nitrocellulose membrane (Bio-Rad, Hercules, CA) and incubated with the specific antibody: DVL-2 (1:500) (Cell Signaling Technology, Beverly, MA), AKT1 (1:1000) (Ser473) (Sigma-Aldrich, St Louis, MO), AKT (1:1000) (Santa Cruz Biotechnology, Santa Cruz, CA), α-TUBULIN (1:1000) (Santa Cruz Biotechnology), GSK-3β (1:500) and pGSK-3b Ser 21/9 (1:500) (Cell Signaling), pβ-CATENIN (Ser33/37/Thr41) and β-CATENIN (both 1:500) (Cell Signaling). Ponceau method to immunoblot was used to ensure equal protein loading [97]. Proteins recognized by antibodies were revealed by ECL technique, following the instructions of the manufacturer (Amersham Biosciences, Piscataway, NJ). To standardize and quantify the immunoblots, we used the photo documentation system DP-001-FDC (VilberLourmat, France) and ImageJ software (US National Institutes of Health, Bethesda, MD; http://rsb.info.nih.gov/ij), respectively. Several exposure times were analyzed to ensure the linearity of the band intensities. α -TUBULIN antibody (Santa Cruz Biotechnology) was used as an internal control of the experiments. Results were expressed in relation to the intensity of α-TUBULIN.

### Nuclear extract

Nuclear extracts of each hippocampus from 4-, 12- and 24-month-old animals were prepared as previously described [98]. Briefly, hippocampal structures were homogenized using a Dounce homogenizer in cold PBS supplemented with 0.5 mM PMSF, 2.5 μg/ml leupeptin and 2.5 μg/ml antipain, and centrifuged at 4°C for 30 sec at 12,000×g. The supernatants (cytoplasmic extract) were reserved for immunoblotting, and the pellets were resuspended in lysis buffer (10 mM HEPES pH 7.9, 1.5 mM MgCl_2_, 10 mM KCl, 0.1 mM EDTA, 0.5 mM PMSF, 2.5 μg/ml leupeptin, and 2.5 μg/ml antipain) and incubated on ice for 10 min. After addition of NP-40 (10%), samples were vigorously mixed and centrifuged for 30 sec at 12,000×g. Supernatant was discarded, and the pellet was resuspended in extraction buffer (20 mM HEPES pH 7.9, 25% glycerol, 1.5 mM MgCl_2_, 300 mM NaCl, 0.25 mM EDTA, 0.5 mM PMSF, 2.5 μg/ml leupeptin, 2.5 μg/ml antipain), incubated 20 min on ice, and centrifuged for 20 min at 12,000×g at 4°C. The resulting supernatants containing nuclear proteins were stored at −80°C. Protein concentration was determined using the Bio-Rad colorimetric assay [99].

### Electrophoretic mobility shift assay (EMSA)

EMSA for NF-κB was performed by using the gel shift assay kit from Promega (Madison, WI), as described previously [98]. ^32^P-NF-κB double-stranded consensus oligonucleotide probe (5′-AGTTGAGGGGACTTTCC CAGGC-3′; 25,000 cpm) and nuclear extracts (15 μg) were used. DNA–protein complexes were separated by electrophoresis through a 6% nondenaturing acrylamide: bis-acrylamide (37.5:1) gel in 0.53 Tris-borate/EDTA (TBE) for 2 h at 150 V. Gels were vacuum dried and analyzed by autoradiography. For competition experiments, NF-κB and transcription initiation factor II (TFIID; 5′-GCAGAGCA-TATAAGGTGAGGTAGGA-3′) unlabeled double-stranded consensus oligonucleotide was included in 20-fold molar excess over the amount of ^32^P-NF-κB probe to detect specific and nonspecific DNA–protein interactions, respectively. Unlabeled oligonucleotides were added to the reaction mixture 20 min before the radioactive probe. Supershift assays, using antibodies against different NF-κB subunits (p50, and RelA, 1:20 dilution), were also conducted according to the protocol of the manufacturer (Santa Cruz Biotechnology) before the incubation of nuclear extracts with the labeled oligonucleotide. Autoradiographs were visualized with a photo-documentation system DP-001-FDC (Vilber Lourmat, Marne la Vallée, France) and quantified in NIH ImageJ software. Several exposure times were analyzed to ensure the linearity of the band intensities.

### RNA extraction and Real-Time PCR

Total RNA was isolated with Trizol reagent (Invitrogen, Carlsbad, CA) from hippocampus of 4, 12 and 24-month old rats according to the instructions of the manufacturer. To confirm the activation status of WNT canonical signaling pathway along aging process, the *Axin 2* gene expression was measured by quantitative PCR (qPCR) using TaqMan gene expression assay (Applied Biosystems, Carlsbad, CA). The gene expression assay used was *Axin2* (Rn00577441_m1, amplicon length=67 bp, RefSeq: NM_024355.1) and the endogenous control was *Hprt1* (hypoxanthine phosphoribosyltransferase 1, Rn01527840_m1, amplicon length=64 bp, RefSeq: NM_012583.2). Prior to performing the experiments, amplification efficiency curves (6 point dilution series) for the gene expression assays were run. The curves allowed quantitation of cDNA such that amplification efficiencies were similar between the *Axin2* and *Hprt*. Real-time PCR analysis was performed with the 7500 Fast Real-Time PCR System (Applied Biosystems, Foster City, CA). Each reaction (performed in duplicate) included 2 μL of diluted (1:10) cDNA, 6.25 μL of Universal PCR Master Mix (Applied Biosystems), 0.625 μL of TaqMan gene expression assay, and sterile bi-destilled water up to a final volume of 12.5 μL. The real-time PCR thermal cycling conditions included a holding stage at 50°C for 2 min and 95°C for 10 min, and 40 cycles of 95°C for 15 sec, and 60°C for 1 min. The Delta-Delta-Ct method was used to quantify the fold change between the samples.

### Statistics

The data, expressed as mean ± standard error of the mean (S.E.M.) were obtained from three independent experiments. In each experiment, three replicate samples were quantified. Data from mRNA expression were analyzed by Delta-Delta-Ct analysis. Statistical comparisons were made by one-way analysis of variance (ANOVA), followed by Newman–Keuls test. All *p* values <0.05 were considered to reflect a statistically significant difference.

## SUPPLEMENTAL FIGURES


