# Supplementary material for: Age-related neuroinflammation and changes in AKT-GSK-3β and WNT/ β-CATENIN signaling in rat hippocampus
Source: Aging (Albany NY). 2015 Dec 6;7(12):1094–108. doi: 10.18632/aging.100853 (PMC4712335; doi:10.18632/aging.100853)
Supplement: Supplementary file 1 [file aging-07-1094-s001.pdf]

SUPPLEMENTAL FIGURES

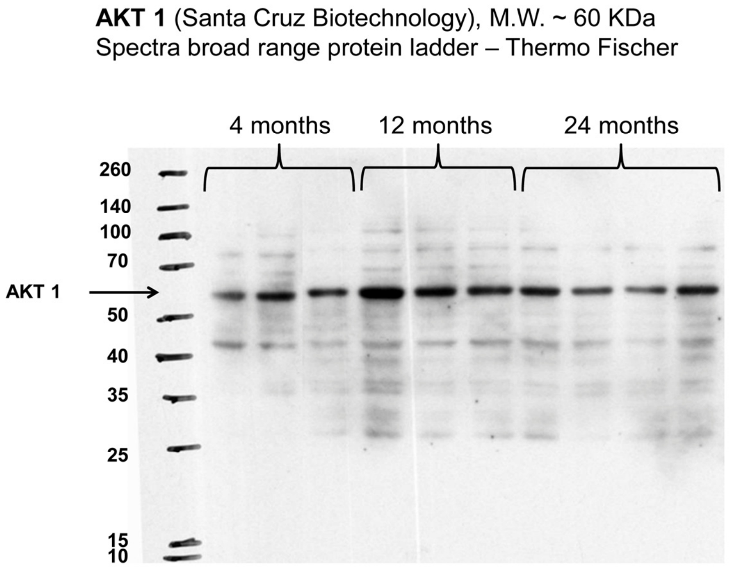

Supplemental Figure 1.

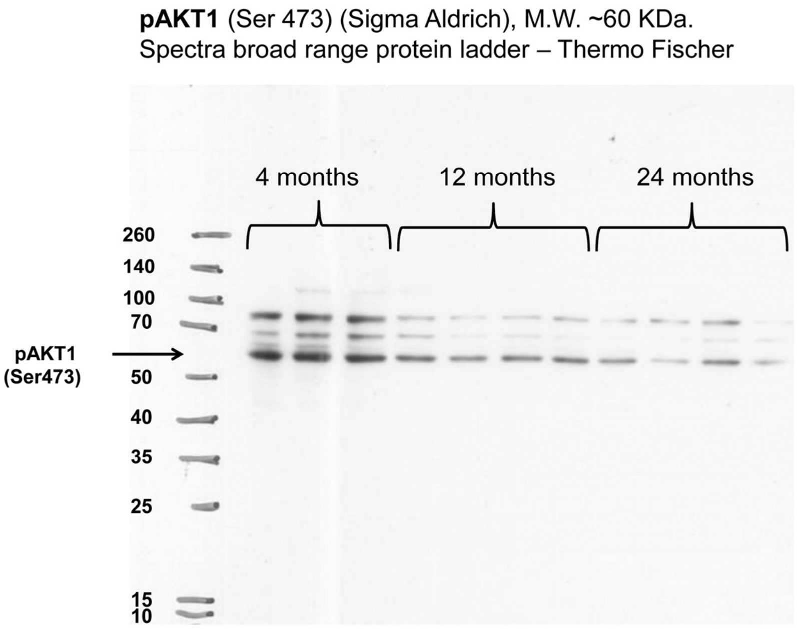

Supplemental Figure 2.

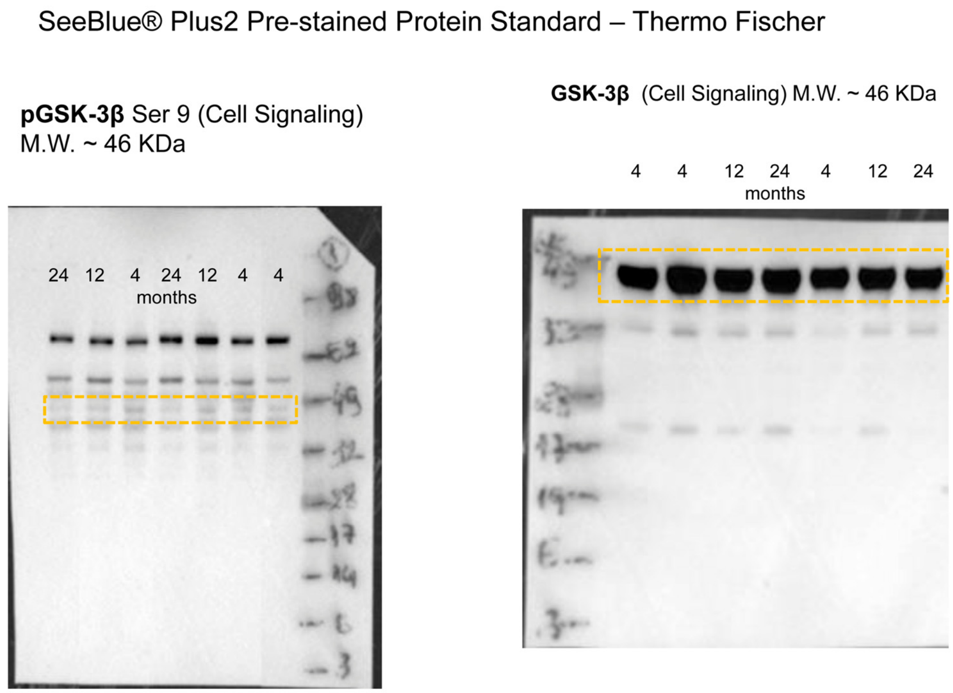

Supplemental Figure 3.

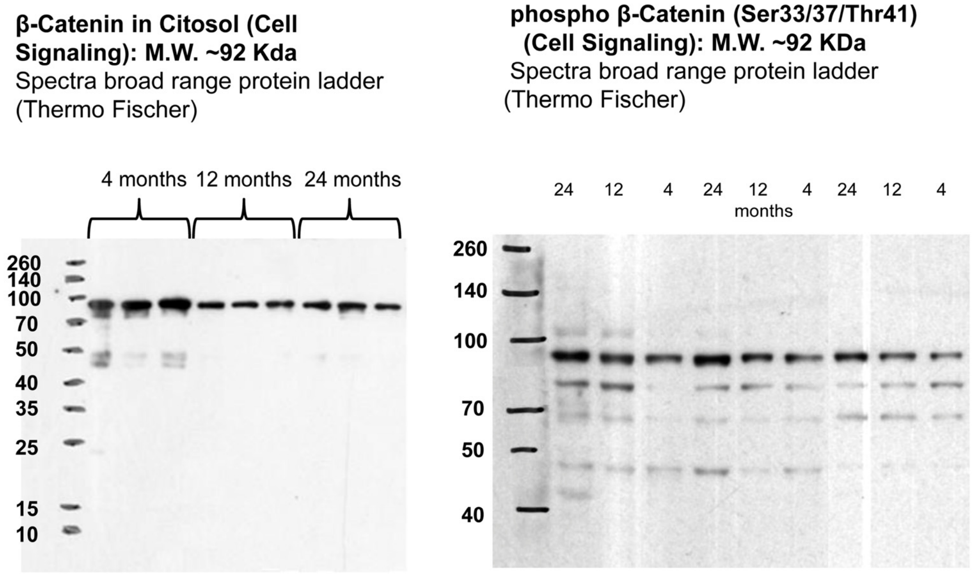

Supplemental Figure 4.

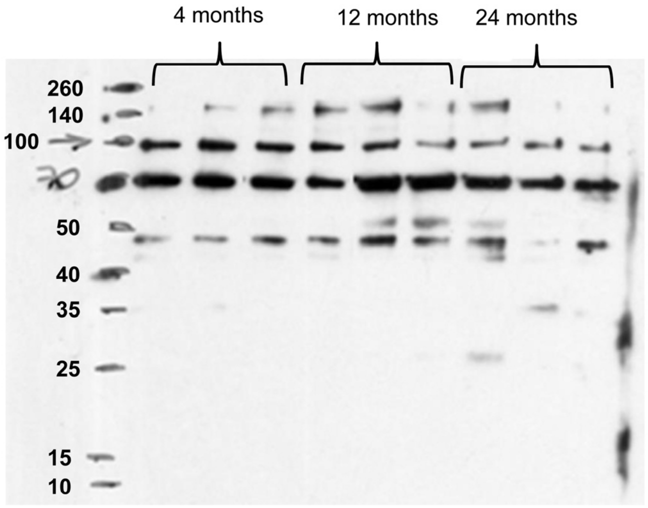

Nuclear β-Catenin (Cell Signaling): M.W. ~92 Kda
Spectra broad range protein ladder (Thermo Fischer)

Supplemental Figure 5.

Dvl-2 (Cell Signaling), M.W ~90-95
HiMark™ Pre-stained Protein Standard, Thermo Fischer

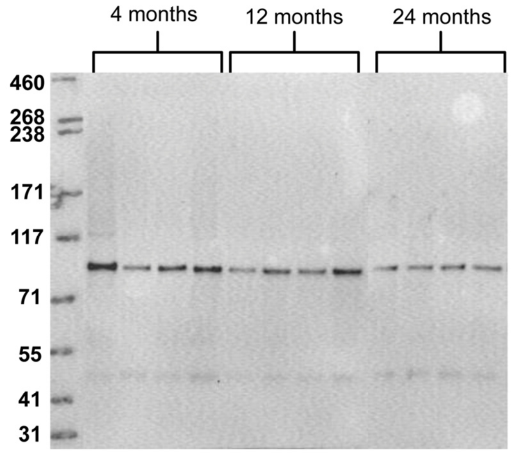

Supplemental Figure 6.
